# Severe falciparum malaria with dengue coinfection complicated by rhabdomyolysis and acute kidney injury: an unusual case with myoglobinemia, myoglobinuria but normal serum creatine kinase

**DOI:** 10.1186/1471-2334-12-364

**Published:** 2012-12-20

**Authors:** Kok Pin Yong, Ban Hock Tan, Chian Yong Low

**Affiliations:** 1 Department of Infectious Diseases, Singapore General Hospital, Outram Road, Singapore, 169608, Singapore

**Keywords:** Falciparum malaria, Rhabdomyolysis, Myoglobinuria, Acute kidney injury

## Abstract

**Background:**

Acute kidney injury (AKI) is a complication of severe malaria, and rhabdomyolysis with myoglobinuria is an uncommon cause. We report an unusual case of severe falciparum malaria with dengue coinfection complicated by AKI due to myoglobinemia and myoglobinuria while maintaining a normal creatine kinase (CK).

**Case presentation:**

A 49-year old Indonesian man presented with fever, chills, and rigors with generalized myalgia and was diagnosed with falciparum malaria based on a positive blood smear. This was complicated by rhabdomyolysis with raised serum and urine myoglobin but normal CK. Despite rapid clearance of the parasitemia with intravenous artesunate and aggressive hydration maintaining good urine output, his myoglobinuria and acidosis worsened, progressing to uremia requiring renal replacement therapy. High-flux hemodiafiltration effectively cleared his serum and urine myoglobin with recovery of renal function. Further evaluation revealed evidence of dengue coinfection and past infection with murine typhus.

**Conclusion:**

In patients with severe falciparum malaria, the absence of raised CK alone does not exclude a diagnosis of rhabdomyolysis. Raised serum and urine myoglobin levels could lead to AKI and should be monitored. In the event of myoglobin-induced AKI requiring dialysis, clinicians may consider using high-flux hemodiafiltration instead of conventional hemodialysis for more effective myoglobin removal. In Southeast Asia, potential endemic coinfections that can also cause or worsen rhabdomyolysis, such as dengue, rickettsiosis and leptospirosis, should be considered.

## Background

Acute kidney injury (AKI) is a feature of severe malaria infection [1], and multiple mechanisms have been implicated [2,3]. Among them, rhabdomyolysis is an uncommon etiology with few reports in the literature [4-8]. The diagnosis is usually based on symptoms of myalgia and high levels of serum muscular enzymes, such as creatine kinase (CK) and myoglobin. Evidence of skeletal muscle damage in falciparum malaria has been found in both children and adults with serum CK and myoglobin concentrations paralleling clinical severity [9-11]. Davis et al. examined the muscle biopsies of 36 cases of falciparum malaria and found parasite sequestration in skeletal muscle and muscle damage, along with a significant correlation between serum CK and myoglobin levels. Even though more than half of the patients suffered AKI with two needing dialysis, only two patients had hemoglobinuria with none having myoglobinuria [11]. In another study involving 12 patients who had severe falciparum malaria (including eight cerebral malaria cases) and elevated CK compatible with rhabdomyolysis, there were no cases with myoglobinuria or hemoglobinuria [8]. Therefore, falciparum malaria complicated by rhabdomyolysis with concomitant raised CK and myoglobinuria is very rare with only two cases reported so far [4,7]. However, to the best of our knowledge, there are no cases of severe falciparum malaria with AKI requiring dialysis as a result of myoglobinuria in the setting of normal CK. In this report, we will detail a case and review the significance of this finding with reference to the selection of appropriate modalities of renal replacement therapy in the management of the AKI.

## Case presentation

A 49-year-old Indonesian man presented with a six-day history of fever, chills, and rigors associated with generalized myalgia that was especially painful over bilateral thighs. This was associated with two episodes of non-bilious, non-bloody vomiting three days before admission and mild dyspnea one day earlier. He had no past medical history, specifically no known renal disease. He was a palm oil plantation worker and had recently traveled to Tanjung Pinang in the Riau Islands two weeks before. On admission, he was febrile at 39.3°C with a pulse rate of 108 beats/min, but normotensive, alert, and oriented. He was dehydrated and jaundiced but did not demonstrate any flapping tremor or pedal edema. There was generalized myalgia with both his thighs visibly swollen and tender to palpation. His heart sounds were dual, lungs were clear, and there was no hepatosplenomegaly on abdominal examination. The neurological examination was also normal. He did not have a rash. Blood samples showed hemoglobin of 17.1 g/dl with hemotocrit of 47.0%, total white blood cell count of 8,910/mm^3^, 8% atypical monocytosis and platelet count of 10,000/mm^3^ (nadir count 6,000/mm^3^). A blood film revealed *Plasmodium falciparum* in 4.0% of erythrocytes. His electrolytes showed moderately severe AKI: urea, 34.2 mmol/l; potassium, 4.4 mmol/l; sodium, 128 mmol/l; creatinine, 550 μmol/l; bicarbonate, 17.1 mmol/l; and glucose, 6.1 mmol/l. His liver function test showed hyperbilirubinaemia of 161 μmol/l and transaminitis with serum ALT 122 U/l, AST 124 U/l, LDH 1,073 U/l, alkaline phosphatase 215 U/l, and albumin 33 g/l. Serum CK was normal at 99 U/l, but aldolase and myoglobin were raised at 17.5 U/l and 138 μg/l (normal 27.8–55.6 μg/l) respectively. Urine analysis revealed myoglobinuria of 194 μg/l (normal <21 μg/l).

**Figure 1:**
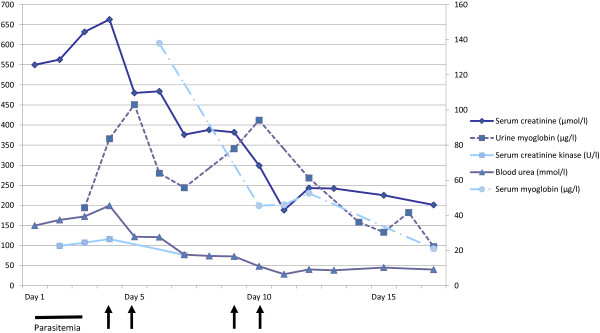
Serum and urine biochemistry changes from time of admission in relation to high-flux hemodiafiltration.

A diagnosis of severe falciparum malaria with rhabdomyolysis and AKI was made, and the patient was treated with intravenous artesunate at 2.4 mg/kg of body weight at 0, 12, and 24 hours, and then once daily for a total of seven days. Concurrent therapy included oral mefloquine 750 mg at 0 hours and 500 mg at 12 hours and intravenous ceftriaxone 2 g once daily. Despite aggressive hydration maintaining good urine output and clearance of parasitemia, the creatinine and urea worsened to 686 μmol/l and 45.5 mmol/l, respectively, on day four. The patient remained acidotic with a bicarbonate nadir of 12.9 mmol/l. His myoglobinuria peaked at 451 μg/l, while serum CK remained normal. Alternative etiologies of rhabdomyolysis were excluded with negative *Mycoplasma pneumoniae* PCR, *Chlamydia pneumoniae* PCR, Leptospira IgM, and *Orientia tsutsugamushi* IgG. *Rickettsia typhi* IgG returned positive at a titre of 1:256, and the patient was given seven days of doxycycline at 100 mg twice daily. However, no rising titre was detected on the convalescent sample. The dengue IgM had a weak positive result on admission and showed a qualitative rise in titre on paired sera, but was negative for the dengue NS1 antigen, suggesting a possible dengue coinfection. Despite the severe thrombocytopenia, he did not have any bleeding tendencies. He underwent renal replacement therapy via hemodiafiltration from days 5 to 10 of admission. Serum myoglobin and urine myoglobin levels improved (See Figure 1), and he was discharged well back to Indonesia with a creatinine level of 201 μmol/l.

## Discussion

The pathogenesis of AKI in severe malaria is multifactorial, and these factors include various inflammatory mediators, intravascular hemolysis and coagulation, hypovolemia from dehydration, hyperparasitemia, and immune complexes, among others [3]. Rhabdomyolysis is a recognized but uncommon cause [4-8]. Miller et al. observed 58 African children with falciparum malaria and found raised CK and myoglobin in 28% and 45%, respectively, at the time of their admission. There was a significant correlation between CK and myoglobin levels and also the severity of the illness as measured by neurological status. AKI, the most common and serious sequela of muscle damage, was however absent in this age group [9]. Earlier reports involving adults with severe malaria also demonstrated poor correlation between raised serum CK and renal dysfunction. Skeletal muscle necrosis severe enough to cause myoglobinuria and AKI is actually very rare, probably because serum concentrations beyond 15,000 ng/mL are required before myoglobin becomes detectable in urine [12]. De Silva et al. and Sinniah et al. separately reported two 17-year-old boys with severe falciparum malaria complicated by oliguric AKI and myoglobinuria needing dialysis. Both cases had documented skeletal muscle necrosis on histopathology and raised CK on biochemistry. The latter also had a renal biopsy demonstrating renal tubules with myoglobin casts in the lumen and foci of interstitial inflammatory cells, including macrophages and T lymphocytes [7,13].

Our patient had non-oliguric AKI with myoglobinemia, myoglobinuria while maintaining a normal serum CK. We are unable to explain why the CK had remained normal in the setting of rhabdomyolysis since CK levels are the most sensitive indicator of myocyte injury in rhabdomyolysis [14]. Nevertheless, rhabdomyolysis with elevated myoglobin without raised CK has been reported in patients with Duchenne muscular dystrophy and malignant hypertension [15]. Myoglobin-induced renal vasoconstriction, direct nephrotoxicity, and sequestered intravascular parasitized erythrocytes in the renal microvasculature probably caused the AKI. Rowland et al. had suggested in the 1970s that the nephrotoxic potential of myoglobin would be enhanced by hypovolaemia, hypotension, fever, and acidosis, all of which may occur in severe malaria [16].

Myoglobin is a 17.8-kDa iron- and oxygen-binding protein found in muscle tissues. It is freely filtered by the glomerulus and, after endocytosis occurs, enters the tubules, where it is metabolized. When the renal threshold is exceeded, it is visible as reddish-brown urine. Myoglobin is both directly and indirectly toxic to the kidneys, and its removal from the renal circulation is paramount in managing AKI [17]. Earlier attempts to remove myoglobin using plasma exchange met with little success [18,19], as did conventional hemodialysis filters, due to the large molecular mass of myoglobin [17,20]. It has been shown that rapid and effective removal of myoglobin can be achieved via continuous venovenous hemofiltration or hemodiafiltration through the use of super high-flux filters and high volumes of ultrafiltration [21-24]. Naka et al. reported a case of serotonin syndrome complicated by severe rhabdomyolysis and oliguric AKI in which super high-flux hemofiltration was able to achieve greater clearance of myoglobin than conventional hemodialysis [22]. Hutchison et al. subsequently reported two patients with rhabdomyolysis and AKI in which hemodialysis with a super high-flux dialyzer cleared nearly 60% of the serum myoglobin with a single dialysis treatment [23]. These observations have been supported by a very recent case series involving six patients [24]. Based on the evidence, super high-flux hemofiltration appears to be a more appropriate dialysis modality for managing myoglobin-induced AKI. In our case, the patient underwent high-flux hemodiafiltration (Fresenius ARrT Plus) and achieved significant myoglobin clearance, as reflected in the rapid decline of serum and urine myoglobin levels that paralleled the recovery of his renal function. Our case report therefore highlights the importance of ascertaining the predominant cause of AKI in severe malaria, which has an impact on the choice of dialysis.

Rhabdomyolysis has been associated with other infectious diseases, and these should be actively diagnosed and treated as potential coinfections in patients with malaria and rhabdomyolysis. Our patient did not have upper respiratory tract symptoms to suggest influenza viral infection, and negative PCRs ruled out mycoplasma and chlamydia pneumonia. The Thais had reported no uncommon dual infections of falciparum malaria with scrub typhus (15%), murine typhus (23.2%) and leptospirosis (7.7%) [25], all of which could cause rhabdomyolysis. Our patient had negative serology for acute leptospirosis and a non-rising positive paired sera titre to *Rickettsia typhi*, suggesting prior murine typhus infection. This is unsurprising given his work exposure to rodents in the oil palm plantation.

Similarly, dengue infection is endemic in Southeast Asia with frequent cyclical epidemics [26], and dengue shock syndrome could certainly contribute to, if not cause, AKI. Malaria and dengue infection share many similar clinical findings, and a delayed diagnosis of either in a patient with coinfection may lead to fatal complications [27,28]. In a recent study regarding diagnostic techniques and management of dengue and malaria coinfection, all patients with dual infections presented with prolonged fever of more than seven days, myalgia, bleeding manifestations, rash, and anemia [29]. Moreover, according to Vasconcelos et al., the continuous fever caused by arboviral infection can mask the periodic fever associated with malarial parasites [30]. Although coinfections are uncommon, the diagnosis of one should not rule out testing for the other [25,31,32]. In French Guiana, 4.3% of 1,723 consecutive febrile patients presenting to the Emergency Department over a one-year period had malaria and dengue coinfection [31]. Our patient, who had fever, severe thrombocytopenia, atypical monocytosis, a positive dengue IgM and a qualitative rise in titre on paired sera probably had a dengue coinfection. The occurrence of severe thrombocytopenia (less than 5,000/mm^3^) supports the concomitant presence of dengue coinfection as it is uncommon in falciparum malaria, occuring in less than 2% in one series [33]. This reinforces the importance that patients with prolonged fever, myalgia, and thrombocytopenia should be evaluated for both malaria and dengue infection.

## Conclusion

In patients with severe falciparum malaria complicated by AKI, it is important to consider rhabdomyolysis as a contributing aetiology. The absence of raised serum CK alone does not exclude a diagnosis of rhabdomyolysis. Raised serum and urine myoglobin levels could lead to AKI and should be monitored. In the event of myoglobin-induced AKI requiring dialysis, clinicians may consider using high-flux hemodiafiltration instead of conventional hemodialysis for more effective myoglobin removal. In Southeast Asia, potential endemic coinfections that can also cause rhabdomyolysis, such as dengue, rickettsiosis, and leptospirosis, should be actively screened for and managed.

## Consent

Written informed consent was obtained from the patient for the publication of this case report. A copy of the written consent is available for review by the Editor-in-Chief of this journal.

## Abbreviations

AKI: Acute kidney injury; CK: Creatine kinase; PCR: Polymerase chain reaction; PTH: Parathyroid hormone.

## Competing interests

The authors declare that they have no competing interests.

## Authors’ contributions

KPY contributed to the clinical management of the patient and writing of the manuscript. BHT contributed to the clinical management of the patient. CYL contributed to the clinical management of the patient and writing of the manuscript. All authors read and approved the final manuscript.

## Authors’ information

KPY: MB BCh. Medical Officer, Department of Infectious Diseases, Singapore General Hospital, Singapore.

BHT: MBBS, FRCP(UK). Senior Consultant and Head, Department of Infectious Diseases, Singapore General Hospital, Singapore. Associate Professor, Duke-NUS Graduate Medical School, Singapore.

CYL: MBBS, MRCP(UK), MMed(Internal Medicine), FAMS. Consultant, Department of Infectious Diseases, Singapore General Hospital. Adjuct Assistant Professor, Duke-NUS Graduate Medical School, Singapore.

## Pre-publication history

The pre-publication history for this paper can be accessed here:

http://www.biomedcentral.com/1471-2334/12/364/prepub
